# First Attack and Clinical Presentation of Hemiplegic Migraine in Pediatric Age: A Multicenter Retrospective Study and Literature Review

**DOI:** 10.3389/fneur.2019.01079

**Published:** 2019-10-15

**Authors:** Irene Toldo, Francesco Brunello, Veronica Morao, Egle Perissinotto, Massimiliano Valeriani, Dario Pruna, Elisabetta Tozzi, Filomena Moscano, Giovanni Farello, Roberto Frusciante, Marco Carotenuto, Carlo Lisotto, Silvia Ruffatti, Ferdinando Maggioni, Cristiano Termine, Gabriella Di Rosa, Margherita Nosadini, Stefano Sartori, Pier Antonio Battistella

**Affiliations:** ^1^Department of Woman's and Child's Health, Juvenile Headache Centre, University Hospital of Padua, Padua, Italy; ^2^Unit of Biostatistics, Epidemiology and Public Health, Department of Cardiologic, Vascular, Thoracic Sciences and Public Health, University of Padova, Padua, Italy; ^3^Division of Neurology, Headache Centre, Children's Hospital Bambino Gesú, Rome, Italy; ^4^Unit of Pediatric Neurology and Epileptology, “Brotzu” Hospital, Cagliari, Italy; ^5^Child Neuropsychiatry Unit, Juvenile Headache Center, University of L'Aquila, L'Aquila, Italy; ^6^Child Neuropsychiatric Unit, Women, Children and Adolescents Health Department, University Hospital S.Orsola-Malpighi, Bologna, Italy; ^7^Pediatric Clinic, Department of Life, Health and Environmental Science, University of L'Aquila, L'Aquila, Italy; ^8^Headache Centre, Bambino Gesù Children's Hospital, Rome, Italy; ^9^Clinic of Child and Adolescent Neuropsychiatry, Department of Mental Health, Physical and Preventive Medicine, University of Campania “Luigi Vanvitelli”, Naples, Italy; ^10^Headache Centre, Pordenone, Italy; ^11^Neurology Unit, South Padua United Hospitals, Padua, Italy; ^12^Department of Neurosciences, Headache Centre, University Hospital of Padua, Padua, Italy; ^13^Child Neuropsychiatry Unit, Department of Medicine and Surgery, University of Insubria, Varese, Italy; ^14^Child Neuropsychiatry Unit, Department of Human Pathology of the Adult and Developmental Age, University Hospital “G. Martino”, Messina, Italy

**Keywords:** hemiplegic migraine, FHM, SHM, children, adolescents

## Abstract

**Background:** Data on clinical presentation of Hemiplegic Migraine (HM) are quite limited in the literature, particularly in the pediatric age. The aim of the present study is to describe in detail the phenotypic features at onset and during the first years of disease of sporadic (SHM) and familial (FHM) pediatric hemiplegic migraine and to review the pertinent literature.

**Results:** Retrospective study of a cohort of children and adolescents diagnosed with hemiplegic migraine, recruited from 11 Italian specialized Juvenile Headache Centers. Forty-six cases (24 females) were collected and divided in two subgroups: 32 SHM (16 females), 14 FHM (8 females). Mean age at onset was 10.5 ± 3.8 y (range: 2–16 y). Mean duration of motor aura was 3.5 h (range: 5 min−48 h). SHM cases experienced more prolonged attacks than FHM cases, with significantly longer duration of both motor aura and of total HM attack. Sensory (65%) and basilar-type auras (63%) were frequently associated to the motor aura, without significant differences between SHM and FHM. At follow-up (mean duration 4.4 years) the mean frequency of attacks was 2.2 per year in the first year after disease onset, higher in FHM than in SHM cases (3.9 vs. 1.5 per year, respectively). A literature review retrieved seven studies, all but one were based on mixed adults and children cohorts.

**Conclusions:** This study represents the first Italian pediatric series of HM ever reported, including both FHM and SHM patients. Our cohort highlights that in the pediatric HM has an heterogeneous clinical onset. Children present fewer non-motor auras as compared to adults and in some cases the first attack is preceded by transient neurological signs and symptoms in early childhood. In SHM cases, attacks were less frequent but more severe and prolonged, while FHM patients had less intense but more frequent attacks and a longer phase of active disease. Differently from previous studies, the majority of our cases, even with early onset and severe attacks, had a favorable clinical evolution.

## Background

Hemiplegic migraine (HM) is a rare form of migraine with aura characterized by transient motor weakness or hemiparesis (motor aura), associated with other non-motor aura symptoms (visual, sensory, aphasic, or basilar-type/brainstem symptoms) accompanied by headache, nausea, vomiting, photophobia, or phonophobia, as occurs in migraine (1). The presence of motor deficits represents the peculiarity of HM compared to other forms of migraine with aura and its diagnostic criteria have been recently revised (ICHD-III, 2018) (2). HM can be sporadic (SHM) or familial (FHM) with autosomal dominant inheritance (1). Three genes have been classically associated with the disease: *CACNA1A, ATP1A2*, and *SCN1A* (1). HM can also be part of the phenotypic manifestations associated with *PRRT2* gene mutations, but *PRRT2* most likely acts as a disease-modifying gene within the context of complex polygenic rather than autosomal dominant disease (3).

The onset of disease is typically in the second decade of life, but it can occur in people aged 1–45 (1). Most of the series available in literature include both pediatric and adult HM cases (4–7) and in these works the pediatric data cannot be extrapolated; series reporting exclusively pediatric cases are very limited (8).

Information on first hemiplegic attack, trigger factors, associated symptoms, duration, and frequency of the attacks and time courses of the disease are lacking, especially in pediatric cases.

## Aim of the Study

The present study describes a large multicenter Italian pediatric cohort of HM in order to refine the clinical phenotype at disease onset and the disease course in children and adolescents affected by HM, through in-depth analysis of personal data and literature review.

## Methods

### Subjects

This was a retrospective study of a cohort of all children and adolescents diagnosed with HM according to the ICHD-III criteria (2), recruited from 11 Italian specialized Juvenile Headache Centers.

The study included all children and adolescents meeting the following entry criteria:

1. diagnosis of HM (according to the ICHD-III criteria) (2);
2. onset of HM before 16 years of age.

The Headache Centers shared clinical criteria and methods related to the study. All the physicians who took part to the study have a consolidated clinical experience in the diagnosis of HM according to the ICHD-III criteria (2) and in its management.

An *ad-hoc* clinical report form was fulfilled for each patient, based on clinical documentation (such as emergency care records, neurological examination records at first visit and during follow-up).

Cases with uncertain diagnosis or incomplete clinical data were excluded.

### Clinical Data

The following clinical data we collected: demographic data, family history for headache or epilepsy, information on first HM attack (including trigger factors, prodromal phase, duration and types of aura, associated signs and symptoms, features and duration of headache, time to complete recovery), personal history for other types of headache or epilepsy, neurological examination in the acute phase and during follow-up, instrumental investigations, genetic tests, symptomatic, and preventive pharmacological therapies of HM.

In our study “total duration of the attack” refers to the duration of the aura symptoms and the headache phase, while “total recovery” represents the time needed to fully return to the condition before the attack onset.

Patients were divided into two subgroups based on the HM family history (SHM or FHM). Clinical data were collected in a database by the leading Center (Padua). The patients' records were handed with respect of confidentiality, and the study protocol was approved by the Institutional Review Board of the Department of Woman's and Child's Health of Padua. The study was conducted according to good clinical practice recommendations of the local Ethics Committee.

### Literature Review

For this update we searched the Pubmed database from the first available paper up to date 1st September 2018. The following search keys were applied: “hemiplegic migraine,” “FHM,” “SHM,” or “HM series.”

The filter was set to English publications.

Three authors (FB, IT, VM) screened all abstracts and full texts available and searched manually for relevant articles. Within the available articles, we searched for adult and pediatric series of HM cases describing clinical and genetic features of patients. Works based on at least 10 cases of HM were selected.

The following exclusion criteria were applied: (1) series formed by <10 patients; (2) series in which clinical features of patients were not available; (3) studies which described clinical and genetic features of one or few (<5) families with FHM.

Genetic and clinical data acquired from each study were reported in Tables F1–F3, S1–S3, M1–M3. FHM series (Tables F1–F3), SHM series (Tables S1–S3), and mixed (FHM+SHM) series (Tables M1–M3) were treated separately.

From each study we searched and reported the following data if available: gender, age of HM onset, clinical features of HM attacks at onset and during follow-up (trigger factors, duration of motor aura, characteristics of non-motor aura), frequency and severity of HM attacks and other associated neurological manifestations, genetic tests.

### Statistical Analysis

Data collected were analyzed through simple descriptive, bi-variate and multivariate analysis. Quantitative variables were summarized by performing continuous descriptive analysis: mean, median, standard deviation, minimum value, and maximum value. If the variables were not normally distributed, location was analyzed by calculating median, first quartile, third quartile. Categorical variables were described by means of absolute and relative frequencies. Where applicable, Chi square test and Fisher exact test were applied to prove a statistical significance of the difference between the frequencies, while the significance of the difference between mean values was evaluated by one-way analysis of variance. Simple and multivariate logistic regression models were applied to assess efficacy and tolerability of the pharmacological and non-pharmacological therapies in relation to type of headache, gender, and age.

In all the tests performed, the level of significance was set at 0.05. The statistical analysis was performed by means of SAS versions 9.13 (SAS Institute, Inc., Cary, North Carolina, USA).

## Results

### General Characteristics of the Study Population

The most relevant general clinical data have been summarized in Table 1 considering the entire population and patients divided in the two subgroups (SHM and FHM).

**Table 1:** Characteristics of the study population.

**Population characteristics**		**TOT (*N* = 46)**	**SHM (*N* = 32)**	**FHM (*N* = 14)**	***p***
**Sex**, F: M (%)		24 (52%): 22 (48%)	16 (50%): 16 (50%)	8 (57%): 6 (43%)	–
**Age at onset** y, mean ± SD (range)		10.5 ± 3.8 (2–16)	10.7 ± 3.7 (2–16)	10.2 y ± 4.2 (4–15)	–
	≤ 6 y	10 (22%)	6 (19%)	4 (29%)	0.46^*^
	7–12 y	18 (39%)	13 (41%)	5 (36%)	–
	13–18 y	18 (39%)	13 (41%)	5 (36%)	–
**Genetics**					
*CACNA1A*	(*n* = 15)	5/15 (33%)	5/13 (38%)	0/2f (0%)	0.52^*^
*ATP1A2*	(*n* = 13)	9/13 (69%)	4/7 (57%)	2/3f (5/6) (66%)	–
*SCN1A*	(*n* = 1)	1/1 (100%)	1/1 (100%)	–	–
*PRRT2*	(*n* = 2)	1/2 (50%)	0/1 (0)	1/1f (100%)	–
**Family history**					
Epilepsy		7/46 (15%)	2/32 (6%)	2/10f (4/14) (20%)	0.23^*^
Migraine°		21/46 (46%)	16/32 (50%)	3/10f (5/14) (33%)	0.30^*^
**Other neurological manifestations**					
Tension-type headache		7/46 (15%)	3/32 (9%)	4/14 (29%)	0.54^*^
Migraine with typical aura		5/46 (11%)	5/32 (16%)	0/14 (0%)	0.30^*^
Migraine without aura		13/46 (28%)	8/32 (25%)	5/14 (36%)	0.49^*^
Febrile seizures		5/46 (11%)	4/32 (13%)	1/14 (7%)	–
Epilepsy		2/46 (4%)	1/32 (3%)	1/14 (7%)	0.52^*^
Cerebellar ataxia		1/46 (2%)	1/32 (3%)	0/14 (0%)	0.23^*^
Intellectual disability		4/35 (11%)	3/23 (13%)	1/12 (8%)	–

*F, females; M, males; f, families: as it concerned genetic data and family history we considered families instead of single patients in FHM subgroups; SD, standard deviation; y, year; –, p > 0.99*;

° *migraine with non-motor aura or without aura*;

* *significance level obtained by Fisher exact test*.

The study population included 46 patients (females *n* = 24, 52%) with pediatric HM. 32/46 (70%) cases were diagnosed as SHM (females *n* = 16, 50%), and 14/46 (32%) cases were diagnosed as FHM (females *n* = 8, 57%). The mean age at first HM attack was similar in both subgroups (SHM 10.7 ± 3.7 y, range 2–16 y vs. FHM 10.2 ± 4.2 y, range 4–15 y; *p* = n.s). 10/46 (22%) cases had the first HM attack before age 6 (SHM 6/32, 19% vs. FHM 4/14, 29%; *p* = 0.46) (Table 1).

Concerning the personal history for other types of migraine, migraine with non-motor aura prevailed in SHM than in FMH cases (SHM 5/32, 16% vs. FHM 0/14, 0%) while migraine without aura was less frequent in SHM than in FHM cases (SHM 8/32, 25% vs. FHM 5/14, 36%). The personal history for febrile convulsive seizures was similar among the two groups (SHM 4/32, 13% vs. FHM 1/14, 7%). One FHM case had a past history of benign myoclonic epilepsy. One SHM patient had a neurological disorder prior to the onset of HM attacks (congenital cerebellar ataxia with bilateral atrophy); this patient, previously described (7), carried a missense mutation c.4013C>T of *CACNA1A* gene.

A higher proportion of SHM patients had a positive family history for migraine (SHM 50% vs. FHM 33%; *p* = 0.33), while a positive family history for epilepsy was more common in FHM cases (SHM 6% vs. FHM 20%; *p* = 0.23) (Table 1).

Genetic tests for HM were performed overall in 24/46 (52%) cases (Table 1). Molecular analysis of *CACNA1A*, performed overall in 15/46 cases, revealed a pathogenic mutation in 5/13 (38%) SHM and in 0/2 (0%) FHM patients. The *ATP1A2* analysis, performed in 13/46 cases, was positive in 4/7 (57%) SHM and in 5/6 (83%) FHM patients. Mutation in the *SCN1A* gene was found in one SHM case, while mutation in the *PRRT2* gene was found in one FHM case (Table 1).

### The First HM Attack

The features of the first HM attack of all patients have been summarized in Table 2.

**Table 2:** Features of the first HM attack in the study population.

**First HM attack**		**TOT (*N* = 46)**	**SHM (*N* = 32)**	**FHM (*N* = 14)**	***p***
**Trigger factors** *at least one trigger*		21/46 (46%)	14/32 (44%)	7/14 (50%)	–
*Emotional stress*		9/21 (43%)	6/14 (43%)	3/7 (43%)	
*Head trauma*		5/21 (24%)	3/14 (21%)	2/7 (29%)	
*Physical effort*		4/21 (19%)	3/14 (21%)	1/7 (14%)	
*Fever*		1/21 (5%)	1/14 (7%)	0/7 (0%)	
*Others*		2/21 (10%)	1/14 (8%, videogames)	1/7 (14%, viral infection)	
**Type of non-motor aura**					
*At least one type*		40/46 (87%)	28/32 (88%)	12/14 (86%)	–
*Visual*		14/40 (35%)	8/28 (29%)	6/12 (50%)	0.30^*^
*Sensory*		26/40 (65%)	16/28 (57%)	10/12 (83%)	0.16^*^
*Aphasic*		4/40 (10%)	3/28 (11%)	1/12 (8%)	–
*Basilar-type (dysarthria, vertigo, etc.)*		25/40 (63%)	16/28 (57%)	9/12 (75%)	0.48^*^
*Number of type simultaneously*	0	6/46 (13%)	4/32 (13%)	2/14 (14%)	–
	1	17/46 (37%)	15/32 (47%)	2/14 (14%)	0.05*
	2	17/46 (37%)	11/32 (34%)	6/14 (43%)	0.74^*^
	3	6/46 (13%)	2/32 (6%)	4/14 (29%)	0.06^*^
**Duration of motor aura**, m or h, mean ± SD (range)		3.5 h ± 8.7 h (5 m−48 h)	4.8 h ± 10.1 h (5 m−48 h)	27.7 m ± 22.7 m (5 m−90 m)	0.001^**^
**Duration of headache**, m or h, mean ± SD (range)		13.7 h ± 17 h (10 m−48 h)	17.0 h ± 18.3 h (20 m−48 h)	5.6 h ± 9.2 h (10 m−24 h)	0.02^**^
**Total duration of HM attack^°^**, m or h, mean ± SD (range)		15.8 h ± 18.9 h (30 m−48 h)	19.6 h ± 20.5 h (40 m−48 h)	6 h ± 8.9 h (30 m−24 h)	0.007^**^
**Signs and symptoms associated**					
*At least one sign or symptom*		29/46(63%)	23/32 (72%)	6/14 (43%)	0.06
*Irritability-agitation*		9/29 (31%)	9/23 (39%)	0/6 (0%)	0.14^*^
*Drowsiness*		7/29 (24%)	5/23 (22%)	2/6 (33%)	0.61^*^
*Vigilance loss*		10/29 (34%)	6/23 (26%)	4/6 (66%)	0.14^*^
*Fever*		7/29 (24%)	7/23 (30%)	0/6 (0%)	0.29^*^
*Seizures*		1/29 (3%)	1/23 (4%)	0/6 (0%)	–
*Others*		3/29 (10%)	3/23 (13%: vomiting, amnesia)	0/6 (0%)	–
*Number of signs/symptoms simultaneously*	0	17/46 (37%)	9/32 (28%)	8/14 (57%)	0.06^*^
	1	21/46 (46%)	15/32 (47%)	6/14 (43%)	–
	>2	8/46 (17%)	8/32 (25%)	0/14 (0%)	0.08^*^
**Prolonged attack**					
Total recovery^°°^>72 h		3/34 (9%)	3/25 (12%)	0/9 (0%)	0.55^*^
Hemiplegia >6 h		3/38 (8%)	3/26 (12%)	0/12 (0%)	0.54^*^
Aphasia >6 h		2/38 (5%)	0/26 (0%)	2/12 (17%)	0.09^*^

*m, minutes, h, hours*;

* *significance level obtained by Fisher exact test*;

** *significance level obtained by Wilcoxon rank sum non-parametric test; –, p > 0.99*;

° *total duration of HM attack refers to the duration of the aura symptoms and the headache phase*;

°° *total recovery represents the time needed to fully return to the condition before the attack onset*.

#### Trigger Factors

21/46 (46%) patients reported at least one trigger factor at the first HM attack (SHM 44% vs. FHM 50%) (Table 2). Emotional stress was the most common trigger factor, reported overall by 43% of patients, without significant differences between SHM and FHM subgroups. Head trauma (SHM 21% vs. FHM 29%) and intense physical effort (SHM 21% vs. FHM 14%) were the other major trigger factors. Fever, viral infections or excessive use of video games were reported in some cases (Table 2).

#### Non-motor Auras

40/46 (87%) cases experienced at least one non-motor aura associated to the motor deficit in the first HM attack, without significant differences between SHM and FHM subgroups; different types of non-motor aura were more frequently reported by FHM than SHM patients (Table 2).

Sensory aura was the most frequent type of non-motor aura (65%), followed by basilar-type/brainstem (63%), and visual (35%) auras. All these three aura types prevailed in FHM patients. On the other side, aphasic aura was slightly more common in the SHM subgroup (11% vs. FHM 8%) (Table 2).

#### Duration of HM Attack

The mean duration of motor aura was significantly longer in SHM subgroup (4.8 h vs. FHM 27.7 min; *p* < 0.01) as well as the mean headache duration (17 h vs. FHM 5.6 h; *p* = 0.02). The mean total duration of the HM attack was over three times longer in SHM than FHM patients (19.6 h vs. 6 h, respectively; *p* < 0.01) (Table 2).

#### Associated Signs and Symptoms

29/46 (63%) patients presented other signs and symptoms associated with the HM attack, more commonly in SHM (23/32, 72%) than FHM patients (6/14, 43%; *p* = 0.06). Vigilance loss (SHM 26% vs. FHM 66%) and drowsiness (SHM 22% vs. FHM 33%) tended to prevail in FHM patients, but without significant differences probably due to sampling number. Irritability associated to episodes of psychomotor agitation and fever were reported only by SHM patients (39 and 30%, respectively) (Table 2).

#### Prolonged Attack

3/34 (9%) patients had a prolonged HM attack lasting at least 72 h as well as a prolonged motor aura (longer than 6 h) and they were all SHM. Two patients had prolonged aphasia (longer than 6 h) and they all were FHM (Table 2).

### Follow-Up

The most interesting data obtained at follow-up have been summarized in Table 3.

**Table 3:** Characteristics of the study population during follow-up.

**Follow-up**			**TOT (*N* = 44)**	**SHM (*N* = 30)**	**FHM (*N* = 14)**	***p***
**Age at follow-up** y, mean ± SD (range)			14.9 ± 6.7 (6–47)	13.9 ± 4.3 (6–27)	17.3 ± 10.5 (6–47)	0.16
**Duration of follow-up** y, mean ± SD (range)			4.4 ± 2.4 (2–38)	3.0 ± 1.6 (2–15)	7.6 ± 4.6 (3–38)	**0.04**
**Frequency of HM attacks (n/y)**						
	First year		2.2 ± 5.3	1.5 ± 4.5	3.9 ± 6.7	0.14^**^
	Later years	No	20/44 (46%)	16/30 (53%)	4/14 (29%)	0.19^*^
		<1/y	10/44 (23%)	5/30 (17%)	5/14 (38%)	0.25^*^
		1/y	6/44 (14%)	4/30 (13%)	2/14 (14%)	–
		>1/y	8/44 (18%)	5/30 (17%)	3/14 (21%)	0.70^*^
**Prolonged HM attacks**						
	Total recovery^°^>72 h		3/46 (7%)	3/32 (9%)	0/14 (0%)	0.54^*^
	Hemiplegia >6 h		7/46 (15%)	6/32 (19%)	1/14 (7%)	0.41^*^
	Aphasia >6 h		2/46 (4%)	1/32 (3%)	1/14 (7%)	0.52^*^
**Alterations revealed by clinical investigations**						
	Acute cytotoxic cortical edema (MRI)		6/46 (7%)	5/32 (10%)	1/14 (0%)	–
	Cerebellar atrophy (MRI)		2/46 (2%)	2/32 (3%)	0/14 (0%)	–
	Slow-wave activity (EEG)		19/29 (66%)	16/23 (70%)	3/6 (50%)	0.88^*^

*y, years; SD, standard deviation; n/y, number per year; h, hours*;

* *significance level obtained by Fisher exact test*;

** *significance level obtained by Wilcoxon rank sum non-parametric test; –, p > 0.99*;

° *total recovery represents the time needed to fully return to the condition before the attack onset*.

Mean duration of follow-up was 4.4 years in the overall population and was longer in FHM patients (7.6 y vs. SHM 3 y; *p* = 0.04).

In the first year after disease onset, the mean number of HM attacks was 1.5 in SHM patients, and 3.9 in FHM patients (*p* = 0.14) (Table 3). Since the second year of disease, mean frequency of attacks was 1–2 every year without significant differences within the two subgroups (SHM 17% vs. FHM 21%; *p* = 0.99). At last follow-up, freedom from attacks in the previous 3 years was reported in 9/19 (47%) SHM patients and in 4/12 (33%) FHM cases (*p* = n.s.).

Similar to the first HM attack, even during the follow-up prolonged attacks were experienced only by SHM patients (3/32, 9% vs. FHM 0/14, 0%; *p* = 0.54) (Table 3).

Neurologic examination at follow-up was abnormal in 3 SHM (2/3 clumsiness, 1/3 hemiparesis), and in 2 FHM patients (1/2 cerebellar ataxia, 1/2 clumsiness).

Cognitive and neuropsychological evaluation was available for 35 patients (23/32 SHM and 12/14 FHM). Among the 23 SHM patients tested, three had mild intellectual disability, one expressive language disorder, one praxis difficulties, and one dyslexia. Among the 12 FHM patients tested the following problems were detected: moderate intellectual disability (1/12), praxis difficulties (1/12), and low sustained attention (1/12).

Preventive drugs (in particular flunarizine and topiramate) were used overall by 9/46 (20%) patients (SHM 5/32, 16% vs. FHM 4/14, 28%).

All patients underwent neuroimaging testing during acute or post-acute phase (CT or MRI); 6 out of 46 cases (13%) showed cortical unilateral (or partial) cytotoxic edema. Two *CACNA1A* mutated patients showed cerebellar atrophy signs (1/2 pancerebellar atrophy with clinical signs of congenital ataxia, 1/2 hypoplasia of superior vermis folia with no clinical signs). Electroencephalogram showed asymmetrical slow-wave activity in 19/29 patients (66%), during or soon after HM attacks.

### Literature Review

A review of the literature retrieved seven studies (3–9) investigating HM series and addressing clinical characteristics (3–9), correlation with genetics (6–11) and features of the HM attacks (4–7). The most interesting results have been summarized in Tables F1–F3 (FHM series), S1–S3 (SHM series), M1–M3 (mixed FHM and SHM series). Any additional selection criteria or clarification about data collection or analysis, concerning each study included in the review, has been highlighted in each table.

All studies but one were based on mixed adults and children cohorts, without a systematic stratification of results by age. The study by Riant et al. (8) reported a cohort of children and adolescents with SHM.

We underline that in all previous studies information about the features of HM attack refers to the “typical HM attack” among the pool of HM attacks experienced by each patient during his/her entire lifespan, while our study provides information specifically on the first HM attack.

## Discussion

The present study, focused on the clinical presentation of HM in an Italian pediatric cohort, shows a certain heterogeneity of the disease onset. Furthermore, both at the onset and during the follow-up, phenotypic differences emerged between the sporadic and the familial form. Moreover, differently from previous literature data, the neurological outcome was favorable in the majority of patients, even in those with early disease onset and severe HM attacks.

Finally, our literature review shows that previous works have not analyzed in detail the clinical onset of HM so far. In the present study, we described for the first time the clinical features of the first HM attack in a series of both SHM and FHM pediatric patients.

### SHM vs. FHM

#### First Attack

At onset, SHM patients tend to have less non-motor auras and more associated signs and symptoms (agitation, drowsiness, alteration of awareness, fever etc.) than FHM patients, which otherwise tend to simultaneously exhibit several non-motor auras but fewer associated symptoms.

Literature dealing with HM is still debating whether awareness impairment should be considered part of the brainstem aura symptoms (3–5) or an associated symptom of the HM attack (6). In our study, we decided to consider this symptom separately in order to give greater emphasis to it and because its duration could exceed the temporal criteria (5–60 min) typical of non-motor auras' symptoms.

Prolonged attack lasting more than 72 h were documented in 32% of SHM cases by Riant et al. (8), while this figure corresponds only to 9% of our SHM cases. However, our SHM patients (particularly those with mutation of one disease genes), had longer duration of hemiplegic attack, motor aura, and headache compared to FHM patients.

In the literature, studies comparing clinical features of SHM with those of FHM are lacking, because SHM and FHM series (based on adults or both adults and children) have been described so far only separately. However, in these studies (see Tables F2, S2), a prolonged duration of motor aura has been reported in SHM cases (5), rather than in FHM cases (4).

In two FHM studies, head trauma was a trigger factor in 25% (6) and 9% (4) of cases, respectively; data on other trigger factors were not reported (Table S2). Riant et al. (8) described 25 SHM pediatric patients reporting the number of HM attacks and their trigger factors during the first years of the disease since onset. In this study, the major trigger factor was head trauma (24%) similarly to our SHM patients (21%). In our cohort a significant proportion of patients (46%) had at least one trigger factor, emotional stress being the most common (43%), reported by Riant et al. only in 8% of cases (8). The occurrence rate and features of trigger factors preceding the first HM attack did not significantly differ between our SHM and FHM patients.

#### Follow-Up

The frequency of hemiplegic attacks ranges from 3 to 4 attacks per year to daily attacks; some patients can experience only one or few episodes in lifetime (1). In our cohort, we found that in the first year of disease the mean frequency of attacks was higher in FHM patients. SHM patients, despite having longer and more severe attacks compared to FHM, tended to have a lower frequency of episodes, especially during the first years after disease onset. On the other hand, the “active phase” of the disease was longer in our FHM cases. However, as far as concerns duration and frequency of attacks, the distribution of values was not normal in the two subgroups, supporting the idea of a wide clinical heterogeneity of the disease both in SHM and FHM cases.

Prolonged attacks can also occur during disease evolution. Indeed we previously reported the case of a girl with SHM, carrying a missense mutation of *ATP1A2*, who presented at age 8 a severe HM attack lasting 6 weeks; in this case the disease begun very early, at age 2 (12).

### Children vs. Adults

In about one sixth of patients (15%), early transient neurological symptoms (i.e., prolonged aphasia during fever, isolated seizures, transient hemiparesis, prolonged clumsiness after minor head trauma) occurred between age 1 and 4 and preceded, even for a long time, the very first episode of HM. These symptoms may be interpreted as isolated auras without a cephalalgic phase or may correspond to the actual disease onset, which is misunderstood because of the child's difficulty in communicating the symptoms. In any case, these findings support the idea that HM can start very early in childhood with non-specific paroxysmal motor or non-motor manifestations. These paroxysmal manifestations should be further investigated, especially in cases with family history for HM.

In our cohort the F:M ratio was about 1, similarly to what reported by Riant et al. (8) in SHM pediatric patients (1.3), while in adults the ratio was much more higher (2.5–6) (4, 7) (Tables F1, M1). It is noteworthy that in adult series this ratio seems to be inversely related to the frequency of the monogenic HM form; in fact the F:M ratio was higher (6:1) in the Hiekkala's series (7) where only 4% of patients had a disease-associated mutation, whereas in the Thomsen's (4) FHM series with a greater frequency of HM monogenic forms, the ratio was 2.5 (Table F1).

It is therefore possible that in the polygenic forms of HM the role of hormones is preponderant, as occurs in the common forms of migraine (13), while in the monogenic forms of HM the role of genetics exceeds that of hormones. Studies on larger populations are necessary to obtain conclusive data.

In our cohort, the prevalence of non-motor auras (visual, sensitive, and aphasic) was remarkably lower than that reported in adults (4–7) (Tables S2, F2, M2). These differences might depend on data collection bias, incomplete recall of the aura at first HM attack in adults, children's inability to describe all the aura symptoms, and on actual change of aura characteristics with age.

In the previous HM series, data on neurological and neuropsychological profile were quite heterogeneous and biased by the study selection criteria and the genetic characteristics of the study population (3–9). In fact, progressive cerebellar ataxia and epilepsy were more frequent in patients with a disease-associated mutation (6, 8) (Tables F3, S3, M3). This figure has been recently confirmed by Pelzer et al. (3).

Among 25 SHM pediatric patients, all carrying a disease-associated mutation, Riant et al. (8) documented a high prevalence of neurological manifestations (epilepsy, ataxia, mental retardation), thus delineating a severe neurological profile in patients with pediatric onset of monogenic HM. However, these patients were recruited from the genetic laboratory, therefore this cohort could not be considered representative of the natural history of pediatric HM due to selection bias and small sample size.

Our cohort, including both patients with and without disease-associated mutation, shows that pediatric HM is associated with cognitive and/or neurological manifestations only in a minority of cases and that the overall neurological outcome is favorable. In fact, during follow-up, none of our patients developed cerebellar signs. Even SHM cases with early disease onset and severe attacks had a favorable clinical evolution.

Limitations of the present study include: (a) the retrospective data collection might have hampered the phenotypic characterization. The recruitment in Centers with a high experience and a multidisciplinary approach should have decreased the underestimation of correct diagnosis and clinical data; (b) the limited number of cases in the two subgroups (SHM and FHM) has failed to achieve, in some analyzes, statistical significance, however the disease is rare and this represents the larger Italian HM population ever reported; (c) genetic analysis was performed overall in half of cases and it is not possible to infer more information about genotype-phenotype correlations, although a maximal effort was made to collect samples to complete genetic analysis.

In conclusion, to the best of our knowledge, this study represents the first Italian pediatric series of HM ever reported, including both FHM and SHM patients.

Our cohort highlights that in the pediatric HM has a heterogeneous clinical onset.

Children present fewer non-motor auras as compared to adults and, in some cases, the first attack is preceded by transient neurological signs and symptoms in early childhood. The overall neurological outcome is favorable in the majority of cases.

A better understanding of the phenotype and natural history of the HM may help identifying prognostic factors, contribute to the genotype-phenotype correlations and guide genetic analysis.

Further multicenter studies on pediatric patient populations are needed in order to evaluate the characteristics of the disease at this age. Finally, studies focused on the neuropsychological profile of these patients are warranted.

## Ethics Statement

All procedures performed in the studies involving human participants were in accordance with the ethical standards of the institutional and/or national research committee and with the 1964 Helsinki Declaration and its later amendments or comparable ethical standards.

## Author Contributions

IT: study concept and design, analysis and interpretation of data, and drafting of the manuscript. FB and VM: acquisition of data, material support, and drafting of the manuscript. EP: statistical analysis and interpretation of data. MV, DP, ET, FMo, GF, RF, MC, CL, SR, FMa, CT, GD: acquisition of clinical data. MN: drafting of the manuscript and English language advice. SS: study supervision. PB: critical revision of the manuscript for important intellectual content.

### Conflict of Interest

The authors declare that the research was conducted in the absence of any commercial or financial relationships that could be construed as a potential conflict of interest.

## Abbreviations

ATP1A2: ATPase Na+/K+ transporting subunit alpha 2
CACNA1A: calcium voltage-gated channel subunit alpha1 A
CT: computed tomography
FHM: familial hemiplegic migraine
HM: Hemiplegic migraine
ICHD-III: International Classification of Headache Disorders third edition
MRI: magnetic resonance imaging
PRRT2: proline rich transmembrane protein 2
SCN1A: sodium voltage-gated channel alpha subunit 1
SHM: sporadic hemiplegic migraine.
