# Supplementary material for: First Attack and Clinical Presentation of Hemiplegic Migraine in Pediatric Age: A Multicenter Retrospective Study and Literature Review
Source: Front Neurol. 2019 Oct 15;10:1079. doi: 10.3389/fneur.2019.01079 (PMC6803542; doi:10.3389/fneur.2019.01079)
Supplement: Supplementary file 1 [file Table_1.doc]

**Table F1-3: FHM series reported in the literature so far and comparison with our cohort.**

**Table F1**: Clinical and genetic characteristics of the major FHM cohorts.

| **Author, year** | **No. of patients (families)** | **Pop.** | **Mean age of onset**  **(range)** | **F:M ratio** | **Occurrence of other types of migraine** | | **Genetic testing** | | | |
| --- | --- | --- | --- | --- | --- | --- | --- | --- | --- | --- |
|  |  |  |  |  | **With aura** | **Without aura** | **CACNA1A** | **ATP1A2** | **SCNA1A** | **PRRT2** |
| Ducros, 2001* | 104  (19) | A (na) + C (na) | 11.7  (1-51) | 1:1 | 9/104  (9%) | 24/104  (23%) | 19/19f  (100%) | / | / | / |
| Thomsen, 2002** | 147  (44) | A (na) + C (na) | 17  (1-45) | 2.5:1 | 80/147  (54%) | 39/147  (26%) | 3/44f  (7%) | 3/44f  (7%) | 0/44f  (0%) | / |
| Present study | 14  (10) | C (14) | 10.2  (4-15) | 1.3:1 | 0/14  (0%) | 5/14  (36%) | 0/2f  (0%) | 2/3f  (66%) | / | / |

**Legend**: A=adults; C=pediatric cases; F=female; M=male; f=families; na=data not available; *this study included 104/104 CACNA1A-mutated patients; ** genetic data concerning this cohort were published in 2007 by the same author.

**Table F2**: Features of the first HM attack in the major FHM cohorts.

| **Author, year** | **Trigger factors** | | | | **Mean duration of motor aura (range)** | **Non motor auras** | | | |
| --- | --- | --- | --- | --- | --- | --- | --- | --- | --- |
|  | **Emotional stress** | **Physical effort** | **Head trauma** | **Others** |  | **Visual aura** | **Sensitive aura** | **Aphasic aura** | **Brainstem aura symptoms** |
| Ducros, 2001* | Most frequent | / | 10/104 (10%) | 2/104° (2%) | 60 m | 66/89  (74%) | 90/97  (93%) | 73/88  (83%) | / |
| Thomsen, 2002 | / | / | 13/147 (9%) | / | 5 h 36 m | 131/147  (89%) | 144/147  (98%) | 106/147  (72%) | 69%§ |
| Present study | 3/14  (21%) | 1/14  (7%) | 2/14  (14%) | 1/14°°  (7%) | 28 m | 6/14  (43%) | 10/14  (71%) | 1/14  (7%) | 9/14  (64%) |

**Legend**: m=minutes; h=hours; *this study included 104/104 CACNA1A-mutated patients; °conventional angiography; °° viral infection; §the author reported the percentage of patients fulfilling BAM criteria of ICHD-1.

**Table F3**: Frequency and severity of HM attacks and other associated neurological manifestations reported in the major FHM cohorts.

| **Author, year** | **Mean duration of the attack**  **(range)** | **Mean number of attacks (range)** | **Severe attacks** | | | **Associated neurological signs and symptoms** | | |
| --- | --- | --- | --- | --- | --- | --- | --- | --- |
|  |  |  | **Complete recovery >72h** | **Loss of awareness** | **Seizures** | **Epilepsy** | **Intellectual disability** | **Ataxia** |
| Ducros, 2001* | na  (30 m – 5 d) | 2-3/y | 38/104  (36%) | 20/104  (19%) | 3/104  (3%) | 0/104  (0%) | / | 62/104 (60%) |
| Thomsen, 2002 | 4 h – 12 h  (30 m - >3 d) | / | 3/147  (3%) | 2/147  (1%) | / | 10/143  (7%) | / | 10/143  (7%) |
| Present study | 6 h  (30 m – 24 h) | 1-2/y  (1-36) | 0/9  (0%) | 0/14  (0%) | 0/14  (0%) | 1/14  (7%) | 1/14  (7%) | 0/14  (0%) |

**Legend**: m=minutes; d= days; h=hours; y=year; na=data not available * this study included 104/104 CACNA1A-mutated patients.
