# Supplementary material for: First Attack and Clinical Presentation of Hemiplegic Migraine in Pediatric Age: A Multicenter Retrospective Study and Literature Review
Source: Front Neurol. 2019 Oct 15;10:1079. doi: 10.3389/fneur.2019.01079 (PMC6803542; doi:10.3389/fneur.2019.01079)
Supplement: Supplementary file 2 [file Table_2.doc]

**Table S1-3: SHM series reported in the literature so far and comparison with our cohort.**

**Table S1**: Clinical and genetic characteristics of the major SHM cohorts.

| **Author, year** | **No. of patients** | **Pop.** | **Mean age of onset**  **(range)** | **F:M ratio** | **Occurrence of other types of migraine** | | **Genetic testing** | | | |
| --- | --- | --- | --- | --- | --- | --- | --- | --- | --- | --- |
|  |  |  |  |  | **With aura** | **Without aura** | **CACNA1A** | **ATP1A2** | **SCNA1A** | **PRRT2** |
| Thomsen, 2003* | 105 | A (na) + C (na) | 16 (M) 21 (F)  (1-44) | 4,3:1 | 62/105  (59%) | 30/104  (29%) | 4/100  (4%) | 4/100  (4%) | / | / |
| De Vries, 2007** | 39 | A (20) + C (19) | 19  (4-42) | / | / | / | 1/39  (3%) | 5/39  (13%) | 1/39  (3%) | / |
| Riant, 2010 | 25 | C (25) | 7.7  (1-15) | 1,3:1 | 2/25  (8%) | 5/25  (20%) | 8/25  (32%) | 11/25  (44%) | / | / |
| Present study | 32 | C (32) | 10.6  (2-16) | 1:1 | 5/32  (16%) | 8/32  (26%) | 5/15  (33%) | 4/7  (57%) | / | / |

**Legend**: A=adults; C=pediatric cases; F=female; M=male; f=families; na= data not available; *genetic data concerning this cohort were published in 2008; **all patients included in this cohort presented a pure form of HM, without other neurological manifestation by definition.

**Table S2**: Features of the first HM attack in the major SHM cohorts.

| **Author, year** | **Trigger factors** | | | | **Mean duration of motor aura (range)** | **Non motor auras** | | | |
| --- | --- | --- | --- | --- | --- | --- | --- | --- | --- |
|  | **Emotional stress** | **Physical effort** | **Head trauma** | **Others** |  | **Visual aura** | **Sensitive aura** | **Aphasic aura** | **Brainstem aura symptoms** |
| Thomsen, 2003 | / | / | / | / | 7 h 5 m  (5 m – >24 h) | 96/105  (91%) | 103/105  (98%) | 85/105  (81%) | 76/105  (72%)§ |
| De Vries, 2007* | / | / | 2/39  (5%) | / | na  (5 m – 1 month) | / | / | / | 4/38  (10%) |
| Riant, 2010 | 2/25  (8%) | 2/25  (8%) | 6/25  (24%) | / | / | / | / | / | / |
| Present study | 6/32  (19%) | 3/32  (9%) | 3/32  (9%) | 2/32°  (6%) | 5 h  (5 m – 48 h) | 8/32  (25%) | 16/32  (50%) | 3/32  (9%) | 16/32  (50%) |

**Legend**: m=minutes; h=hours; na= data not available; *all patients included in this cohort presented a pure form of HM, without other neurological manifestation by definition; ° fever, video games; §the author reported the percentage of patients fulfilling BAM criteria of ICHD-1.

**Table S3**: Frequency and severity of HM attacks and other associated neurological manifestations reported in the major SHM cohorts.

| **Author, year** | **Mean duration of the attack**  **(range)** | **Mean number of attacks (range)** | **Severe attacks** | | | **Associated neurological signs and symptoms** | | |
| --- | --- | --- | --- | --- | --- | --- | --- | --- |
|  |  |  | **Complete recovery >72h** | **Loss of awareness** | **Seizures** | **Epilepsy** | **Intellectual disability** | **Ataxia** |
| Thomsen, 2003 | 4 h – 12 h  (30 m - >24 h) | / | 2/105  (2%) | / | / | / | / | / |
| De Vries, 2007* | na  (20 m – 1 month) | na  (0-250) | 6/39  (15%) | 4/39  (10%) | / | 0/39  (0%) | 0/39  (0%) | 0/39  (0%) |
| Riant, 2010 | na  (10 m – 3 weeks) | na  (0-36) | 8/25  (32%) | 5/25  (20%) | / | 12/25  (47%) | 10/25  (40%) | 7/25  (28%) |
| Present study | 18 h 30 m  (40 m – 48 h) | 1-2/y  (0-50) | 3/32  (9%) | 1/32  (3%) | 1/32  (3%) | 1/32  (3%) | 3/22  (14%) | 1/32  (3%) |

**Legend**: m=minutes; h=hours; y=year; na= data not available; *all patients included in this cohort presented a pure form of HM, without other neurological manifestation by definition.
