# Supplementary material for: First Attack and Clinical Presentation of Hemiplegic Migraine in Pediatric Age: A Multicenter Retrospective Study and Literature Review
Source: Front Neurol. 2019 Oct 15;10:1079. doi: 10.3389/fneur.2019.01079 (PMC6803542; doi:10.3389/fneur.2019.01079)
Supplement: Supplementary file 3 [file Table_3.doc]

**Table M1-3: Mixed series (FHM and SHM) reported in the literature so far and comparison with our cohort.**

**Table M1**: Clinical and genetic characteristics of the major mixed (FHM+SHM) cohorts.

| **Author, year** | **No. of patients** | **Pop.** | **Median age of onset** | **F:M ratio** | **Occurrence of other types of migraine** | | **Genetic testing** | | | |
| --- | --- | --- | --- | --- | --- | --- | --- | --- | --- | --- |
|  |  |  |  |  | **With aura** | **Without aura** | ***CACNA1A*** | ***ATP1A2*** | ***SCNA1A*** | ***PRRT2*** |
| Hiekkala, 2018 | 406 | G+ (13)  G- (280) | 12 | 6:1 | / | / | 4/293  (1%) | 9/293  (3%) | / | / |
| Pelzer, 2018* | 208 | G+ (208) | 10 | 1.2:1 | / | / | 107/208  (51%) | 75/208  (36%) | 26/208  (13%) | / |
|  | 73 | G- (73) | 12 | 2.3:1 | / | / | 0/73  (0%) | 0/73  (0%) | 0/73  (0%) | / |
| Present study | 46 | G+ (16)  G- (8) | 11.5 | 1.1:1 | 5/46  (11%) | 13/46  (28%) | 5/15  (33%) | 9/13  (69%) | / | / |

**Legend**: F=female; M=male; f=families; *the author distinguished patients with positive genetic testing results and patients with negative genetic testing results; clinical features of both subgroups were analyzed separately; G+=patients with positive testing results for mutations in *CACNA1A, ATP1A2, SCN1A*; G-=patients with negative testing results for mutations in *CACNA1A, ATP1A2, SCN1A*.

**Table M2**: Features of the first HM attack in the major mixed (FHM+SHM) cohorts.

| **Author, year** | | **Trigger factors** | | | | **Median duration of motor aura** | **Non-motor auras** | | | |
| --- | --- | --- | --- | --- | --- | --- | --- | --- | --- | --- |
|  |  | **Emotional stress** | **Physical effort** | **Head trauma** | **Others** |  | **Visual aura** | **Sensitive aura** | **Aphasic aura** | **Brainstem aura symptoms** |
| Hiekkala, 2018 | | / | / | *3/10°*  *(33%)* | / | / | 68.2% | 99% | 88.6% | / |
| Pelzer, 2018* | G+ | / | / | 59/126 (47%) | / | 60-120 m | 142/162  (88%) | 153/155  (99%) | 104/114 (91%) | 134/208  (64%)§ |
|  | G- | / | / | 2/49  (4%) | / | 60-75 m | 63/68  (93%) | 63/65  (96%) | 41/51  (80%) | 33/73  (45%)§ |
| Present study | | 9/46  (20%) | 4/46  (9%) | 5/46  (11%) | 3/46  (7%) | 60 m | 14/46  (30%) | 26/46  (57%) | 4/46  (9%) | 25/46  (54%) |

**Legend**: m=minutes; h=hours; *the author distinguished patients with positive genetic testing results and patients with negative genetic testing results; clinical features of both subgroups were analyzed separately; G+=patients with positive testing results for mutations in *CACNA1A, ATP1A2, SCN1A*; G-=patients with negative testing results for mutations in *CACNA1A, ATP1A2, SCN1A*; °the frequency of minor head trauma as trigger factor is known only among patients with positive genetic testing results; §the author did not included dysarthria among brainstem aura symptoms.

**Table M3**: Frequency and severity of HM attacks and other associated neurological manifestations reported in the major mixed (FHM+SHM) cohorts.

| **Author, year** | | **Duration of the attacks** | **Mean number of attacks (range)** | **Severe attacks** | | | **Associated neurological signs and symptoms** | | |
| --- | --- | --- | --- | --- | --- | --- | --- | --- | --- |
|  |  |  |  | **Complete recovery >72h** | **Loss of awareness** | **Seizures** | **Epilepsy** | **Intellectual disability** | **Ataxia** |
| Hiekkala, 2018 | | 4-72 h  (75%)° | / | 15% | 16% | / | 3% | / | / |
| Pelzer, 2018* | G+ | / | / | / | / | 11/29  (38%) | 12/82  (15%) | 10/180  (6%) | 26/28  (93%) |
|  | G- | / | / | / | / | 1/6  (17%) | 1/22  (5%) | 0/72  (0%) | 0/4  (0%) |
| Present study | | 6 h  (median) | 1-2/y  (0-50) | 3/46  (7%) | 1/46  (2%) | 1/46  (2%) | 2/46  (4%) | 4/35  (11%) | 1/46  (2%) |

**Legend**: h=hours; y=year; *the author distinguished patients with positive genetic testing results and patients with negative genetic testing results: clinical features of both subgroups were analyzed separately; G+=patients with positive testing results for mutations in *CACNA1A, ATP1A2, SCN1A*; G-=patients with negative testing results for mutations in *CACNA1A, ATP1A2, SCN1A*; °in 75% of cases, the duration of attacks is within 4 and 72 h.
